# Is the reverse vaccinology idea becoming exhausted?

**DOI:** 10.3389/fimmu.2026.1730217

**Published:** 2026-02-05

**Authors:** Javier Zumárraga, Daniel López, Javier Sotillo, Michael J. McConnell, Antonio J. Martín-Galiano

**Affiliations:** 1 Escuela Internacional de Doctorado Universidad Nacional de Educación a Distancia (EIDUNED), Madrid, Spain; 2 Immune Presentation and Regulation Unit, Centro Nacional de Microbiología, Instituto de Salud Carlos III, Madrid, Spain; 3 Parasitology Reference and Research Laboratory, Centro Nacional de Microbiología, Instituto de Salud Carlos III, Madrid, Spain; 4 Department of Biological Sciences, University of Notre Dame, Notre Dame, IN, United States; 5 Proteomics Unit, Core Scientific and Technical Units, Instituto de Salud Carlos III, Madrid, Spain

**Keywords:** adjuvant, antigen selection, antimicrobial resistance, epitope analysis, immunoinformatics, machine learning, pandemics, vaccine licensing

## Abstract

Reverse vaccinology (RV) was originally conceived to leverage genomic information for antigen selection and promised a paradigm change in vaccine design. After a steady increment since 2000 and surge in 2021, RV-related publications have recently plateaued, accompanied by declining journal impact factors and a shift from immunology and microbiology to more technical and general categories. Despite its potential and a favorable data science scenario, the impact of RV on the vaccine portfolio concerning pandemics, antimicrobial resistant pathogens and calendar campaigns remains almost negligible. The lack of multidisciplinary collaboration in many RV studies has led to a predominance of purely theoretical studies without experimental validation, likely contributing to waning interest within the broader vaccinology community. For instance, a growing fraction of RV studies focuses on multi-epitope constructs, which limited successful antecedents make their performance questionable in practice. Additionally, target pathogens are increasingly redundant with existing vaccines or of marginal immediate relevance, further fueling skepticism about RV’s real-world value. This decoupling underscores the need to renew the original idea by integrating RV with complementary frameworks such as systems vaccinology, network vaccinology, and artificial intelligence, as well as embedding RV within higher-order experimental and translational efforts. Furthermore, policymakers and the pharmaceutical sector have relied almost exclusively on classical antigenic elements such as attenuated or inactivated microorganisms, capsular components and fimbria proteins. Importantly, alignment with key stakeholders is essential to bridge early computational insights with late-stage vaccine development. Without this integration to cover the whole vaccine lifecycle, RV risks losing relevance.

## 1 Introduction

Shortly after the sequencing of the first bacterial genome, Rino Rappuoli proposed the original hypothesis of *in silico* antigen discovery, leveraging genomic information for vaccine design in the late 1990s (1). In 2007, he provided proof-of-concept to support this, with a RV-driven trivalent formulation that protects against *Neisseria meningitidis* group B (2), licensed as Bexsero (3, 4). This highlighted that RV was not only a pure ideation but a useful approach that generates true biomedical value.

RV has had considerable promise as an alternative to traditional experimental screening of antigens, offering benefits in cost, time, effort, and safety. Immunoinformatics has substantially reshaped the way antigens are identified through the prediction of B-cell and T-cell epitopes at an unprecedented speed and scale (5). The RV paradigm has been progressively enriched with the incorporation of novel filtering criteria—such as gene essentiality, high sequence conservation, subcellular localization, and predicted off-target effects—and the refinement of pre-existing ones (6). Five technical pillars have further promoted RV, namely, (i) availability of many fully-sequenced genomes for most virulent microorganisms; (ii) three-dimensional information for nearly all proteins (7); (iii) the arousal of a large community of algorithm developers; (iv) the production, standardization and storage of immunological data in accessible resources; and (v) the universalization of supercomputing. As a result, RV pipelines have populated the field of fundamental antigen discovery for more than two decades (8). Consequently, they have been applied to virtually all relevant bacterial and viral pathogens, and extended to some fungi and parasites.

Despite the undeniable contributions of RV to the theoretical vaccine field, the number of licensed immunoprophylactic products that have directly resulted from this strategy remains disproportionately low. This mismatch between hypothetical and practical success has raised concerns across the field. For instance, many RV-predicted antigens fail to induce protective responses *in vivo*, revealing persistent challenges such as low predictive power for conformational epitopes and insufficient capacity to provide immunological context to data (9–11). Furthermore, virtual vaccine design may not have achieved critical data and algorithmic maturity to overcome constraints encountered when modeling the complexity of the desired response to elicit (12). Altogether, these drawbacks affecting RV can misguide research efforts and resource allocation.

In this study, we examine the current state of RV, reflecting on its achievements, limitations, and points of stagnation. Also, we have interrogated the relevance of RV publications besides the molecular nature of the resulting antigens and the selected pathogen types, and interpretated the trends of the outcomes. In addition, we have explored the nature of antigen selection—and design—of current approved vaccines or candidates under evaluation in human trials, and the role of RV in the principal human infectious threats. Finally, we propose potential lines of evolution to guide the next generation efforts of computational vaccinology, including potential meeting points between RV and late vaccine development players.

## 2 Critical RV aspects

### 2.1 Bibliography and scientific impact of RV studies

**Figure 1:**
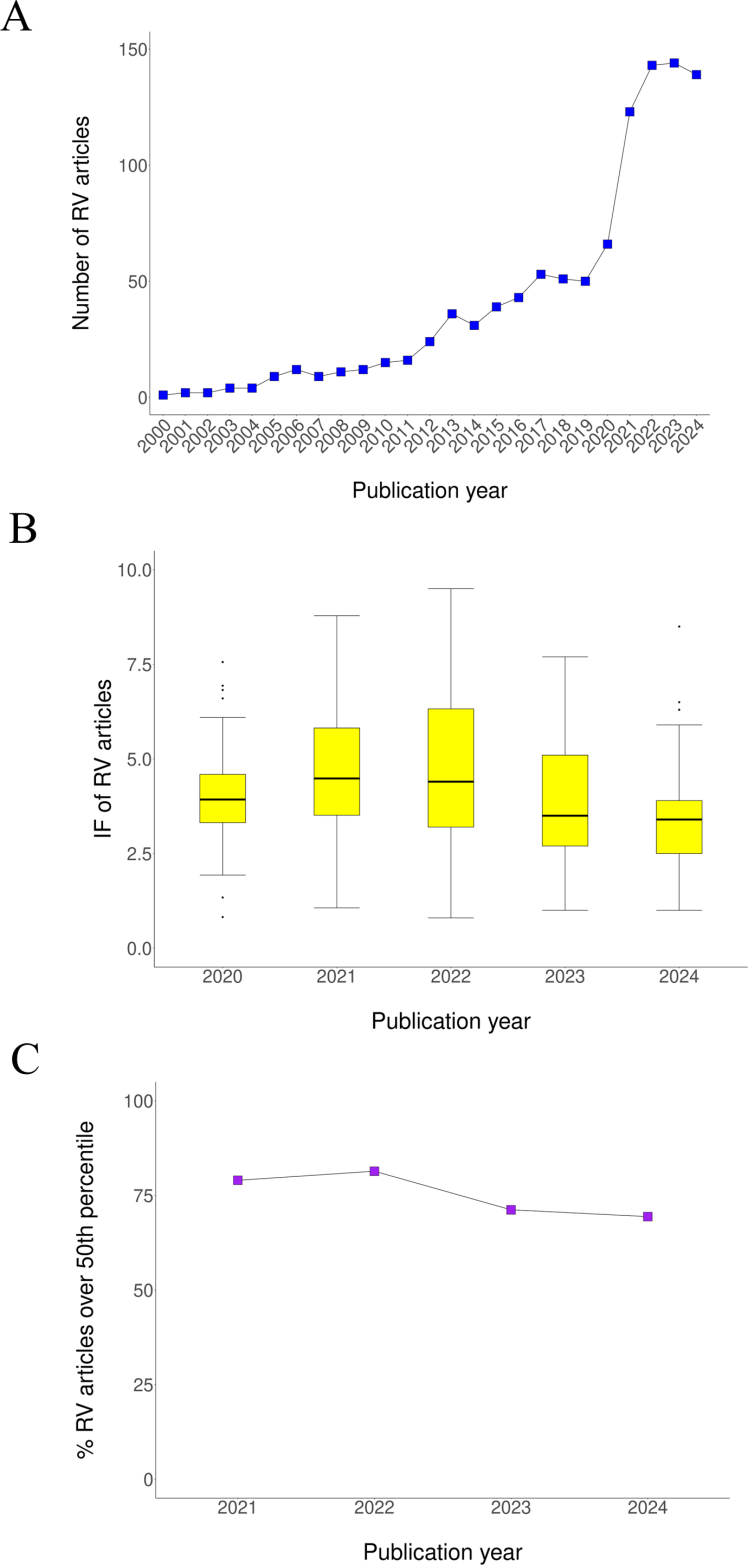
Temporal publication trends of RV articles. **(A)** Total number of RV articles per year in Pubmed. Articles were identified by searching the term “reverse vaccinology”. **(B)** Median and interquartile range of IF values for RV articles by publication year. IF values and scientific area(s) for over 91% MEDLINE-indexed journals publishing RV articles were acquired from Clarivate (https://clarivate.com/). **(C)** Percentage of RV articles published in journals above the 50th percentile of their category. If a journal is assigned to more than one area, only the highest percentile was considered.

The analysis of RV-related bibliography showed an incipient decrease in impact metrics and changes of direction in methodology and target pathogen. As previously observed by others (13), a steady increase in the number of RV-related publications was observed since the early 2000s followed by a pronounced surge in 2021, with 123 publications (**Figure 1A**). This coincided with renewed global interest in vaccine development sparked by the success of COVID-19 vaccine initiatives. Since then, the number of studies has plateaued at around 140 articles/year. More important is that the contemporary impact factor (IF) of journals for these publications progressively dropped since 2021 (**Figure 1B**). Average IF fell from 6.4 to 4.2 and median IF from 4.8 to 3.4 from 2021 to 2024. Simultaneously, the fraction of RV articles in journals over the 50th percentile in their respective scientific area decreased from 79% to 69% (**Figure 1C**).

**Figure 2:**
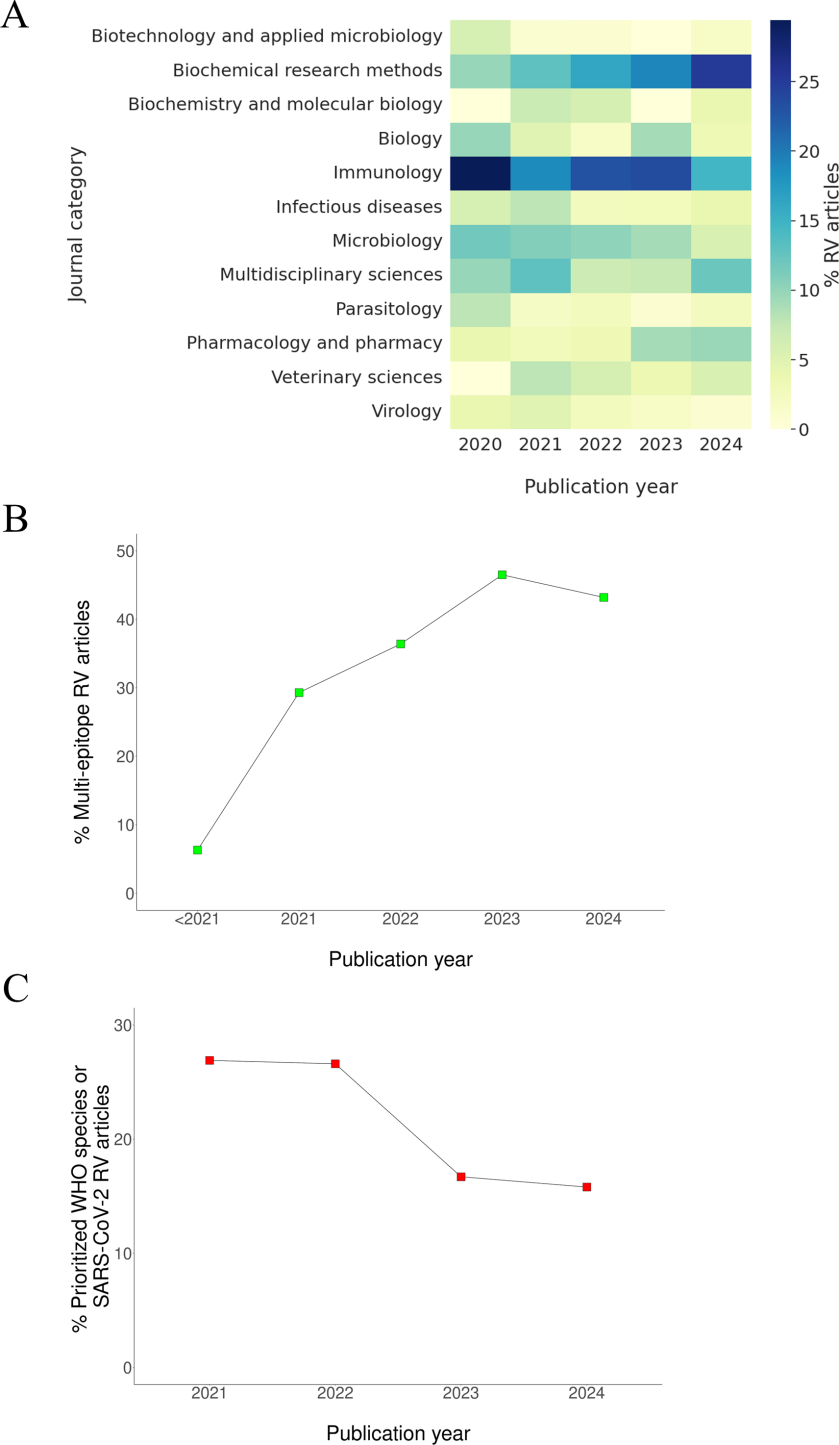
Journal category, multi-epitope strategy and target pathogen statistics of RV articles. **(A)** Percentage of RV articles per year by journal category. Only MEDLINE-indexed journals within the top 12 most RV-prevalent subject areas are shown. **(B)** Percentage of Pubmed publications per year identified utilizing the combined search terms “reverse vaccinology” and “multi-epitope”. **(C)** Percentage of Pubmed publications per year identified utilizing “reverse vaccinology” and each scientific name of the twelve pathogens prioritized by the WHO for antibiotic resistance, or SARS-CoV-2, as combined search terms.

This moderate reduction in top 50th percentile papers, compared to the sharper IF tendency, may indicate changes in the scientific category. In the period 2020-2024, the dominant RV publishing landscape gradually shifted from “Immunology” to “Biochemical research methods” (**Figure 2A**). Other areas such as “Infectious diseases”, “Microbiology”, “Parasitology” or “Virology” were also progressively replaced by “Pharmacology and Pharmacy” and interdisciplinary areas. This may signal that RV is transitioning from a tool tightly adapted to the immunobiology of target pathogens toward a more technical or exploratory approach.

Contrary to protocols established within the principles of biomedicine where *in vivo* verification is mandatory, nearly all RV studies remained exclusively computational and concluded with the mantra “these antigens must be experimentally validated”. This recurring disclaimer, while methodologically honest, may contribute to the perceived disconnect between *in silico* predictions and real-world vaccine development pipelines.

In addition, the fraction of RV studies designing linker-bound multi-epitope constructs as the principal outcome increased from 5% until 2020 to 43% in 2024 (**Figure 2B**). The resulting hybrid antigens are typically modelled, mapped on Ramachandran plots to validate favorable protein folding areas, subjected to molecular dynamics and docked to toll-like receptors (14). These approaches allow for combining the most relevant sections of several antigens and, compared to protein purification, the resulting products are easy to synthesize and inexpensive to scale up industrial production levels. Among disadvantages, the response robustness can be overridden compared to using the original whole antigens. In addition, the barrier against immunological escape by mutants carrying minor genetic changes is also expected to be significantly lower. The multi-epitope idea reached its peak of interest around two decades ago and may still be useful under some specific contexts. However, it was essentially oriented to cellular protection against intracellular viruses (15). In contrast, most bacterial, fungal and parasitic pathogens are extracellular and demand effective humoral protection, which mainly depends on conformational, often discontinuous B-cell epitopes (16), impossible to reproduce here.

Unveiled trends also concerned selection of the target pathogen. These growingly included particular sublineages (17), opportunistic (like for cystic fibrosis patients) (18), veterinary interest (19), very endemic cases—such as Bourbon virus in USA (20), OZ virus in Japan (21) or Langya henipavirus in China (22)—and even non-confirmed yet as virulent species such as *Vandammella animalimorsus* (23). All these pathogens warrant surveillance but, due to their specialization, it is at present unlikely that these studies call the attention of the fundamental drivers of vaccine development, evaluation, and licensing. Reversely, the fraction of RV papers concerning SARS-CoV-2 and multidrug-resistant species prioritized by the WHO decreased from 2021 to 2024 (**Figure 2C**). In 2024, RV-driven antigenic alternatives to other primary pathogens (*Mycobacterium tuberculosis*, *Neisseria meningitidis*, influenza, etc.) were still reported, despite the fact that high-quality antigens in these species are already available.

### 2.2 RV has low impact on licensed and advanced experimental vaccines

The development of a vaccine is a very difficult process. Only a tiny fraction, ca. 5-10%, of vaccine prototypes are eventually licensed for distribution in populations (24–26). This extreme attrition was expected to be mitigated by RV. Based on this assumption, we evaluated the impact of RV designs on vaccines already licensed or in current clinical trials. For that, we manually scrutinized VIOLIN: https://violinet.org/ (27) and https://clinicaltrials.gov/ resources in the absence of consolidated platforms that integrate information on authorized vaccines, those under patent protection, and RV-derived candidates. We focused on three prioritizing epidemiologic scenarios encompassing urgency for vaccine availability: (i) COVID-19 pandemics, (ii) antimicrobial resistance, and (iii) diseases with high prevalence included in standard vaccine calendars.

COVID-19 exemplifies prompt demand of prophylaxis against a pandemic, with seven million confirmed deaths and a 3% reduction of world Gross domestic product (28). While RV provided precise data tunable to strains-of-concern (29, 30), the popular licensed vaccines were based on classical antigenic solutions and developed and applied to populations in the month-range. For viruses with few structural proteins, straightforward development of vaccines and monoclonal antibodies is feasible using the most abundant and exposed protein, *i.e.* the spike of SARS-CoV-2, akin to hemagglutinin in influenza (31) and the fusion protein of the respiratory syncytial virus (32). The spike was further engineered by experts by introducing key prolines that stabilized the prefusion, “locked”, conformation (33), an antigenic improvement that requires deep molecular knowledge rather than RV-like tools. Thus, RV appears more useful for pandemics due to bacteria or to viruses with higher proteomic complexity than for those with compact genomes.

Difficult-to-treat bacterial pathogens are a principal threat to public health, with worrisome mortality and cost projections (28). The list of updated priority pathogens in this respect is periodically listed in WHO reports. Numerous RV studies have targeted these species as they are the leading cause of nosocomial infections, although with varying levels of experimental verification (34). However, most scientific efforts focused on traditional targets and novelty is rather based on platforms, *e.g.* the use of extracellular vesicles, and other stages rather than antigen screenings *in silico* (35). While no vaccine has been licensed for these microorganisms (36), https://clinicaltrials.gov/listed 39 entries in FDA-monitored trials involving WHO-prioritized pathogens, none of which involved previous RV selection, but relied on attenuated strains, capsular constituents, or fimbrial structures. Namely, RV may have re-discovered or refined some of these classical antigens but seemingly not directly participating in the antigen selection role expected of it.

Calendar vaccines mostly involve classical antigenic solutions not related either to sophisticated omic- and algorithm-level investigations. These campaigns are responsible for an estimation of between 97 and 154 million lives saved in the XXI century just before COVID-19 (37, 38). Thus, these are one of the main factors responsible for the extreme reduction of child mortality and increased life expectancy observed worldwide. Among these types of vaccines, the hexavalent vaccine—against diphtheria, tetanus, pertussis, poliomyelitis, *Haemophilus influenzae* type b and hepatitis B—involves toxoids, inactivated microorganisms and recurrent antigens. Measles, mumps, and rubella (MMR vaccine) in addition to chickenpox, influenza and rotavirus formulations also implicate attenuated or inactivated viruses. Prevalent vaccines against human papillomavirus consist of virus-like particles from capsid proteins. Tetravalent meningococcus and novel pneumococcus vaccines include conjugated capsular polysaccharides. This leaves the seminal study on meningococcus B (Bexsero) mentioned above as, to our knowledge, the only RV case in this vaccine category.

## 3 Discussion

Antigen selection via computer simulations emerged almost three decades ago as a postgenomic tool suited for vaccine development. However, this has not delivered a substantial portfolio of effective immunological solutions against the most pressing infectious threats. Thus, RV-like techniques run the risk of being relegated by vaccine experts to purely speculative tools. The misalignment between RV and the development of vaccine end products appears multicausal and due to internal and external reasons. While the former ones mostly relate to limited performance on experimental tests, the later ones are associated with a lack of familiarity among late-stage vaccine developers and a scarcity of computationally emergent precedents. Here, we have attempted to audit the applicability of the RV idea over time, current fundamental limitations and possible strategies to deal with them.

Although secondary reasons may also be involved (e.g. epidemic or editorial board scope changes), an analysis of the explosion of RV reports from 2021 onwards strongly suggests growing weaknesses. Although arguable, these weaknesses very likely hamper the performance of the field and are responsible for the progressive decline in the impact of RV papers and growing skepticism in some areas. These include the recurrent absence of experimental verification, the steady movement to debatable multi-epitope hybrid solutions and the selection of target pathogens with already optimal public health options or with those that hardly will return the investment. Unless RV confront real gaps in vaccine availability, i.e. pathogens with large impact on the population for which there is no vaccines with broad and durable protection, several concerns will persist. These concerns relate to redundancy and the rationale for research for replacing well-established and effective decades-old vaccines in pandemics, multidrug resistance and calendar pathogens.

Mature preclinical vaccine antigen candidates and end products must be tightly adapted to the pathoimmunology of the germs and the expected target population. This requires multidisciplinary expert teams and expensive facilities to obtain key experimental support (39). As a gold illustrative example of RV efficacy, Bexsero development was based on multiple antigen expression in *E. coli*, rodent immunization, and immunological assays. In contrast, most RV cases converged into comparable report-like procedures executed using universal algorithms and data—in some cases without a deep immunological or microbiological experience regarding the pathogen in question—and no functional validation. Thus, the promise of cheap and rapid investigations, can make the RV community to succumb to the temptation of short-term publications, avoiding the risk of costly laborious experiments. However, this is at the expense of generating preliminary results with unknown prophylactic value, compromising the ultimate goal of vaccine development. Thus, RV history missed the opportunity to refine original predictions and distil causal insights, at least, by successive iterations with *in vitro* test or preclinical models. Understandably, simplicity and low cost could contribute to the democratization of early-stage vaccine discovery in environments with limited access to advanced experimental installations. In these cases, international collaboration would facilitate coupling with appropriate validation pipelines potentially uncovering antigens that would otherwise remain unexplored.

Advantages such as design flexibility and immunogenicity breadth explain the RV community drift towards multi-epitope constructs. However, these protocols present important concerns that reduce their biotechnological attractiveness even today (40). The expected improvements resulting from combining epitopes from several antigens are deeply dependent of rules regarding epitope-centered protection, inter-epitope synergy and immunodominance that are far from being understood (41). In contrast, Bexsero, the epitome of successful RV, includes three complete antigen proteins. Admittedly, multi-epitope vaccines warrant a renewed experimental exploration but, to our knowledge, none advanced to further human stages and very few studies involved animal models (42). Thus, the overrepresentation of hybrid schemes with insufficient support in the RV field adds considerable uncertainty to the area and likely contributes to the disconnect between RV scientists and vaccine production lines.

RV is one among a handful of theoretical techniques that perceive rational vaccine design from different angles (43). Several emergent fields of study marginally overlap with RV but also complement it as, for instance, systems vaccinology approaches the host response of the vaccinees in a multi-omic manner (44); network vaccinology aims to model the complex interactions between immune system elements (cells, proteins, metabolites, etc.) using the strength of graph theory (45); and structural vaccinology takes advantage of three-dimensional structures to optimize epitope analysis of antigens (46). Despite the inclusion in RV of the term “vaccinology” (defined as “the science and study of vaccines, encompassing their development, production, immune system response to them, safety, etc.”), in practice RV remains focused on the initial selection and/or design of antigen candidates.

Thus, to reconcile semantics and fully contribute to vaccine development, we believe that RV should be integrated at least with the complementary approaches indicated above. Ideally, this should be carried out under the guidance of multidisciplinary experts and encompassed within a more holistic framework such as “Knowledge-based vaccinology” (KBV). KBV tools should aim to identify optimal combinations of antigens, adjuvants, delivery platforms, dosages, vehicles, and administration regimens, as this is experimentally unapproachable due to the curse of dimensionality (47). KBV should establish intellectual and computational bridges among these categories to enhance their performance beyond what can be achieved when they are considered in isolation. We anticipate an approach analogous to personalized oncology, in which the convergence of genetics, pharmacology and machine learning enables the prediction of effective treatments (48). As the fusion of the involved disciplines is still in its infancy, it is difficult to provide concrete examples of advanced KBV prototypes. Thus, we envisage the KBV roadmap will evolve through a gradual transitional phase to become the standard for rational vaccine design, in which classical RV and higher-order approaches will coexist, rather than resulting from an abrupt conceptual leap.

External factors affecting RV include the tendency of large pharmaceutical companies and public entities to perpetuate successful past antigen types. Attenuated/inactivated microorganisms, polysaccharidic capsular components, in addition to viral spike-like proteins, abundant outer proteins and bacterial fimbrial adhesins nearly monopolize licensed vaccines or those under study in humans. Despite the apparent reductionism, reluctance to utilize non-classical antigenic strategies is grounded in the pressing need at scale to ensure consistent performance and minimize legal, safety, and public cost risks. This clashes with theoretical scientific and publishing pressures of pure RV teams resulting in a hypertrophy of the number of vaccine candidates opening the gap with respect to those probed useful. Consequently, the RV community’s lack of integration into the complete vaccine lifecycle creates a conflict between early-stage and late-stage developers.

To overcome this barrier, the RV groups need to participate and reach a critical mass of real-world antecedents. This trajectory would persuade large companies and public institutions to invest in their outcomes. To balance both worlds, bidirectional awareness initiatives, targeted meetings, and interdisciplinary consortia could emphasize the potential of data science and establish new lines of thinking among project evaluators, R&D directors at pharmaceutical firms and policymakers. In return, these key stakeholders should provide insight concerning epidemiology, social expenses, society damage, legal background, large-scale calculations and organize human cohorts following refined RV suggestions. This would forge a solid generation of vaccine experts that unify the RV strengthens and late challenges. Otherwise, attachment to pre-genomic paradigms surely make governments miss optimal opportunities that remain in the shade and RV initiatives will “die on the shore” without human-testing.

Overall, we suggest RV must extend its focus to experimental validation, encompass the entire vaccine lifecycle and foster closer collaboration with late-stage vaccine development stakeholders. Otherwise, despite its contributions to early antigen selection, RV may be limited in delivering on its promise of transforming the vaccine development pipeline. In any case, we remain optimistic that the knowledge-based approach to vaccine design initiated with RV is merely undergoing growth pains and still has the potential to evolve into a powerful framework to address urgent infectious threats.

## Data Availability

The original contributions presented in the study are included in the article/supplementary material. Further inquiries can be directed to the corresponding author.
